# Disentangling the roles of maternal and paternal age on birth prevalence of down syndrome and other chromosomal disorders using a Bayesian modeling approach

**DOI:** 10.1186/s12874-019-0720-1

**Published:** 2019-04-23

**Authors:** James A. Thompson

**Affiliations:** College of Veterinary Medicine and Biomedical Science, Texas A&M University, College Station, TX 77843-4475 USA

**Keywords:** Paternal age, Maternal age, Down syndrome, Chromosomal disorders, Conditional auto-regressive

## Abstract

**Background:**

Multiple neonatal and pediatric disorders have been linked to older paternal ages. Combining these findings with the evidence that many men are having children at much later ages generates considerable public health concern. The risk of paternal age has been difficult to estimate and interpret because children often have parents whose ages are similar and likely to be confounded. Epidemiologic studies often model the conditional effects of paternal age using regression models that typically treat maternal age as linear, curvilinear or as age-band categories. Each of these approaches has limitations. As an alternative, the current study measures age to the nearest year, and fits a Bayesian model in which each parent’s age is given a conditional autoregressive prior (CAR).

**Methods:**

Data containing approximately 12,000,000 birth records were obtained from the United States Natality database for the years 2014 to 2016. Date were cross-tabulated for maternal ages 15–49 years and for paternal ages 15–65 years. A Bayesian logistic model was implemented using conditional autoregressive priors for both maternal and paternal ages modeled separately and jointly for both Down syndrome and chromosomal disorders other than Down syndrome.

**Results:**

Models with maternal and paternal ages given CAR priors were judged to be better fitting than traditional models. For Down syndrome, the approach attributed a very large risk to advancing maternal age with the effect of advancing paternal age having a very small sparing effect on birth prevalence. Maternal age was also related to the birth prevalence of chromosomal disorders other than Down syndrome while paternal age was not.

**Conclusions:**

Advancing paternal age was not associated with an increase in risk for either Down syndrome or chromosomal disorders other than Down syndrome.

**Electronic supplementary material:**

The online version of this article (10.1186/s12874-019-0720-1) contains supplementary material, which is available to authorized users.

## Background

A rapidly increasing list of neonatal and pediatric disorders has been linked to older paternal ages [1]. Combining these findings with the evidence that many men are having children at much later ages generates considerable public health concern [2, 3]. The best known of the many conditions that have been linked to older paternal age are stillbirths, birth defects, childhood cancers and neurodevelopmental disorders, specifically autism spectral disorders and schizophrenia [1]. However, considerable controversy exists in identifying the conditions caused by paternal age because the analysis needs to adjust for mother’s age [4–7]. There is considerable theory to explain potential associations between neonatal disorders and paternal age. It has long been known that males, with advancing age, have a nonlinear increase in germ-line mutations with age related to cumulative changes with the spermatagonial stem cells [8]. These effects result from age-related changes that compromise DNA replication, DNA repair, cell cycle control, and epigenetic modifications in spermatagonial stem cells and these errors accumulate with successive mitotic divisions [9, 10] and contribute to de novo mutations, affecting genetic traits in a variety of ways [11]. Now, the evidence that paternal germline mutations are responsible for a variety of conditions is considered overwhelming and the list continues to grow [1]. In spite of the perceived magnitude of the problem, the epidemiologic search for the causative mutagens has stalled. Three reviews over a period of 18 years trace the history of the, so far, futile search for mutagens responsible for paternal germline mutations [12–14]. The most important difficulty appears to be confounding, especially the confounding by maternal age and, presumably by maternal exposures.

When modeling the joint effects of maternal and paternal ages, two approaches have predominated [15]. Often, age is measured to the closest year and maternal and paternal ages are modeled as linear or as curvilinear (linear and quadratic). This approach is usually inadequate because the linear and quadratic functions will often fit well over specific age ranges and fit poorly over other age ranges. Furthermore, the best fitting linear and quadratic forms will be dependent upon the scale of the model. For example, the scale for the logistic model is usually log-linear as opposed to linear. The second predominant approach has been to stratify ages into categories which can leave residual confounding within age categories [15]. As an alternative, we propose a Bayesian modeling approach that measures age to the nearest year and models each parent’s age as a conditional autoregressive (CAR) [16]. The CAR prior facilitates smoothing of age-specific parental risk estimates to the risk estimates of ages one year younger and one year older as an autoregressive function. This approach facilitates relatively precise estimation of age-related risk especially under the condition of non-linearity. While joint conditioning of both parental ages is arbitrarily complex, it is straightforward under a Markov Chain Monte Carlo (MCMC) implementation. Under the Bayesian MCMC implementation, the risk estimate for one parent’s age will be adjusted for the full distribution of possible effects of the other parent’s age (not just the mean of the expected risk, for example). The objective of this study was to parse the maternal and paternal age effects on Down syndrome (DS) and chromosomal disorders other than Down syndrome (CD). The novel approach should help resolve the current uncertainty on the direct effects of paternal age on these syndromes. Furthermore, an approach to parsing parenteral age effects for a wide variety of disorders will be illustrated and validated. The approach has potential to provide an advantage to the estimation of the risks of paternal age and, thus, could enable the identification of multiple disorders mediated by mutations during spermatogenesis. Such an advantage may promote the identification of specific cumulative exposures contributing to the causes of age-related paternal risk.

## Methods

### Database

Data containing approximately 12,000,000 birth records were obtained from the United States Natality database for the years 2014 to 2016. In the United States, state laws require birth certificates to be completed for all births, and federal law mandates national collection and publication of births and other vital statistics data. The National Vital Statistics System, the federal compilation of these data, is the result of the cooperation between the National Center for Health Statistics (NCHS) and the states to provide access to statistical information from birth certificates. This study was evaluated by the Texas A&M Institutional Review Board (IRB) and determined to be exempt from IRB review.

### Model 1 – maternal age random-walk (CAR)

For each of DS and CD, case counts were cross-tabulated by j = 35 maternal ages (15 to 49 years). For each row in the table Y_j_ was the count of cases, at birth, and n_j_, the count of births. The counts, Y_j_ were modeled as independent Binomial distributions conditional on an unknown rate parameter (μ_j_).

$$ {\mathrm{Y}}_{\mathrm{j}}\sim \mathrm{Binomial}\ \left({\upmu}_{\mathrm{j}},{\mathrm{n}}_{\mathrm{j}}\right) $$

The logit of the rate parameter was then modeled as a linear function of the overall intercept and a random effect for each maternal age.

$$ \mathrm{Logit}\ \left({\upmu}_{\mathrm{j}}\right)=\alpha +\mathrm{materna}{\mathrm{l}}_{\mathrm{j}} $$

The intercept was given a flat, improper prior. The maternal prior was a minimally informative CAR or random walk prior of length 35 (ages (j) = 15 to 49). The precision of the CAR prior was specified as uniform (0,10) on the standard deviation scale.

### Model 2 – paternal age random-walk (CAR)

For each of DS and CD, case counts were cross-tabulated by k = 51 paternal ages (15 to 65 years). For each row in the table Y_k_ was the count of cases, at birth, and n_k_, the count of births. The counts, Y_k_ were modeled as independent Binomial distributions conditional on an unknown rate parameter (μ_k_).

$$ {\mathrm{Y}}_{\mathrm{k}}\sim \mathrm{Binomial}\ \left({\upmu}_{\mathrm{k}},{\mathrm{n}}_{\mathrm{k}}\right) $$

The logit of the rate parameter was then modeled as a linear function of the overall intercept and a random effect for paternal age.

$$ \mathrm{Logit}\ \left({\upmu}_{\mathrm{k}}\right)=\alpha +\mathrm{paterna}{\mathrm{l}}_{\mathrm{k}} $$

The intercept was given a flat, improper prior. The paternal prior was a minimally informative CAR or random walk prior of length 51 (ages (k) = 15 to 65). The precision of the CAR prior was specified as uniform (0,10) on the standard deviation scale.

### Model 3 – fully conditional random-walk (CAR)

For each of DS and CD, case counts were cross-tabulated by j = 35 maternal ages (15 to 49 years) and k = 51 paternal ages (15 to 65 years). For each row in the table Y_jk_ was the count of cases, at birth, and n_jk_, the count of births. The counts, Y_jk_ were modeled as independent Binomial distributions conditional on an unknown rate parameter (μ_jk_).

$$ {\mathrm{Y}}_{\mathrm{jk}}\sim \mathrm{Binomial}\ \left({\upmu}_{\mathrm{jk}},{\mathrm{n}}_{\mathrm{jk}}\right) $$

The logit of the rate parameter was then modeled as a linear function of the overall intercept and a random effect for each maternal and paternal age.

$$ \mathrm{Logit}\ \left({\upmu}_{\mathrm{j}\mathrm{k}}\right)=\alpha +\mathrm{materna}{\mathrm{l}}_{\mathrm{j}}+\mathrm{paterna}{\mathrm{l}}_{\mathrm{k}} $$

The intercept was given a flat, improper prior. The maternal prior was a minimally informative CAR or random walk prior of length 35 (ages (j) = 15 to 49). The paternal prior was a minimally informative CAR or random walk prior of length 51 (ages (k) = 15 to 65). The precision of both CAR priors was specified as uniform (0,10) on the standard deviation scale.

The implementation allowed a burn-in of 5000 iterations then the next 10,000 iterations were sampled for the posterior distribution. Convergence was evaluated by observing convergence of separate chains with diverse starting values. The median, the lower 2.5% limit and the upper 97.5% limit were all drawn from the complete posterior distributions. The authors refer to the interval from the 2.5 percentile to 97.5 percentile values as the 95% Bayesian credible interval. When the lower bound of this credible interval is greater than 1, the value for the Bayesian exceedance probability would be greater than 95% which would be relatively analogous to a frequentist *p*-value of less than 5% for a 2-tailed test [17]. The CAR prior produces estimates of random effects that sum to zero at the scale of the log odds. For presentation purposes, the CAR estimates were transformed to odds ratios standardized to parental ages of 15 years.

The CAR models were compared to the Bayesian version of more common models including a linear model, a linear and quadratic model that we refer to as curvilinear and a model that divided age into 5-year age categories. The age categories were 15–19, 20–24, 25–29, 30–34, 35–39, 40–44 and 45–49 for each parent’s age and three additional categories for fathers’ ages namely, 50–54, 55–59 and 60–65. Minimally informative Normal priors with zero mean and wide variance, specifically N(0,1000), were used for intercepts, linear, quadratic and age-category effects. To compare final CAR models to these three models, all odds ratios were adjusted to use the overall mean risk as the baseline risk and the medians from the posterior distributions were plotted. Model fit was evaluated using the Deviance Information Criterion (DIC). [18] All models and the data are available in the Additional files 1–6.

## Results

The study identified 11,943,020 births over the three-year period. Of these births, 10,293,589 could be determined have the mother’s age within the 15 to 49-year range and the father’s age belonged in the 15 to 65-year range. Excluded observations included 1,642,373 births for which the father’s ages were not recorded. These observations included all births for which a father was not identified. Exclusions included 324 for which both mother and father were younger than 15 y and 49 births for which both the mother was older than 49 and father was older than 65. In observations for which the mothers’ ages were eligible, the father’s age was greater than 65 for 2539 births and less than 15 y for 450 births. When fathers’ ages were eligible, the mother’s age was greater than 49 y for 1894 births and less than 15 y for 1802. There were no exclusions for either combination of the father older than 65 y and mother younger than 15 y or father younger than 15 y and mother older than 49 y.

The cross-tabulated data (*n* = 10,293,589) included 5390 children with Down syndrome with the diagnosis confirmed for 2273 and listed as probable for 3117 children. A diagnosis is considered “confirmed” after chromosomal evaluation and is considered “probable” when based on clinical signs at birth. Chromosomal disorders other than DS were identified in 4147 children including confirmed for 1349 children and probable for 2798 children.

**Fig. 1:**
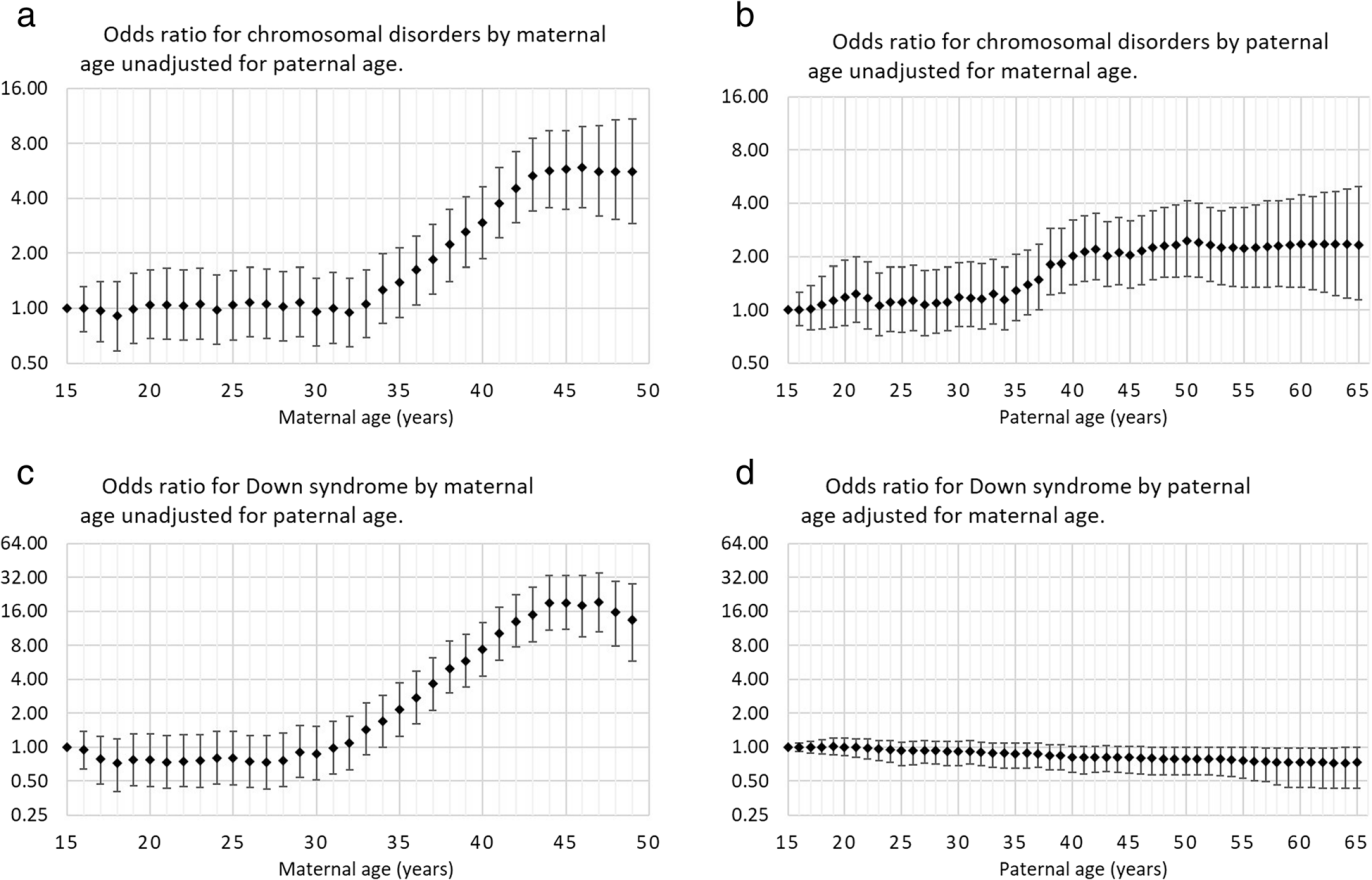
Median odds ratio and 95% Bayesian credible interval, by parental age, relative to age 15 y for Down syndrome for (**a**) maternal age unadjusted for paternal age; (**b**) paternal age unadjusted for maternal age; (**c**) maternal age adjusted for paternal age and (**d**) paternal age adjusted for maternal age

The odds ratio for maternal age, unadjusted for paternal age, started to increase at approximately age 30 and then increased relatively constantly, on the log scale, up to age 45 y, where the odds ratio appeared to stop increasing. The maximum odds ratio relative to 15-year-old women, was approximately 16-fold (Fig. 1a). The odds ratio for paternal age, unadjusted for maternal age, started to increase at approximately age 30 and then increased relatively constantly, on the log scale, up to age 45 y, where the odds ratio appeared to stop increasing. The maximum odds ratio, relative to 15-year-old men, was approximately four-fold (Fig. 1b). The plot of adjusted odds ratios for maternal age was very similar to the plot of the unadjusted odds ratios. When comparing a mother’s age 45 y to age 15 y, the median odds ratio and 95% credibility intervals was 18.9 (11.1, 32.7) for Down syndrome, when adjusted for paternal age (Fig. 1c). For paternal ages, the plot of odds ratios showed the odds ratios to be very near unity but tending to show risk sparing (i.e., odds ratio less than 1). After age 49 y, the odds ratio had a 95% credibility interval that excluded 1. When comparing age 45 y to age 15 y, the median odds ratio and 95% credible interval was 0.81 (0.60, 1.01) for Down syndrome (Fig. 1d).

**Fig. 2:**
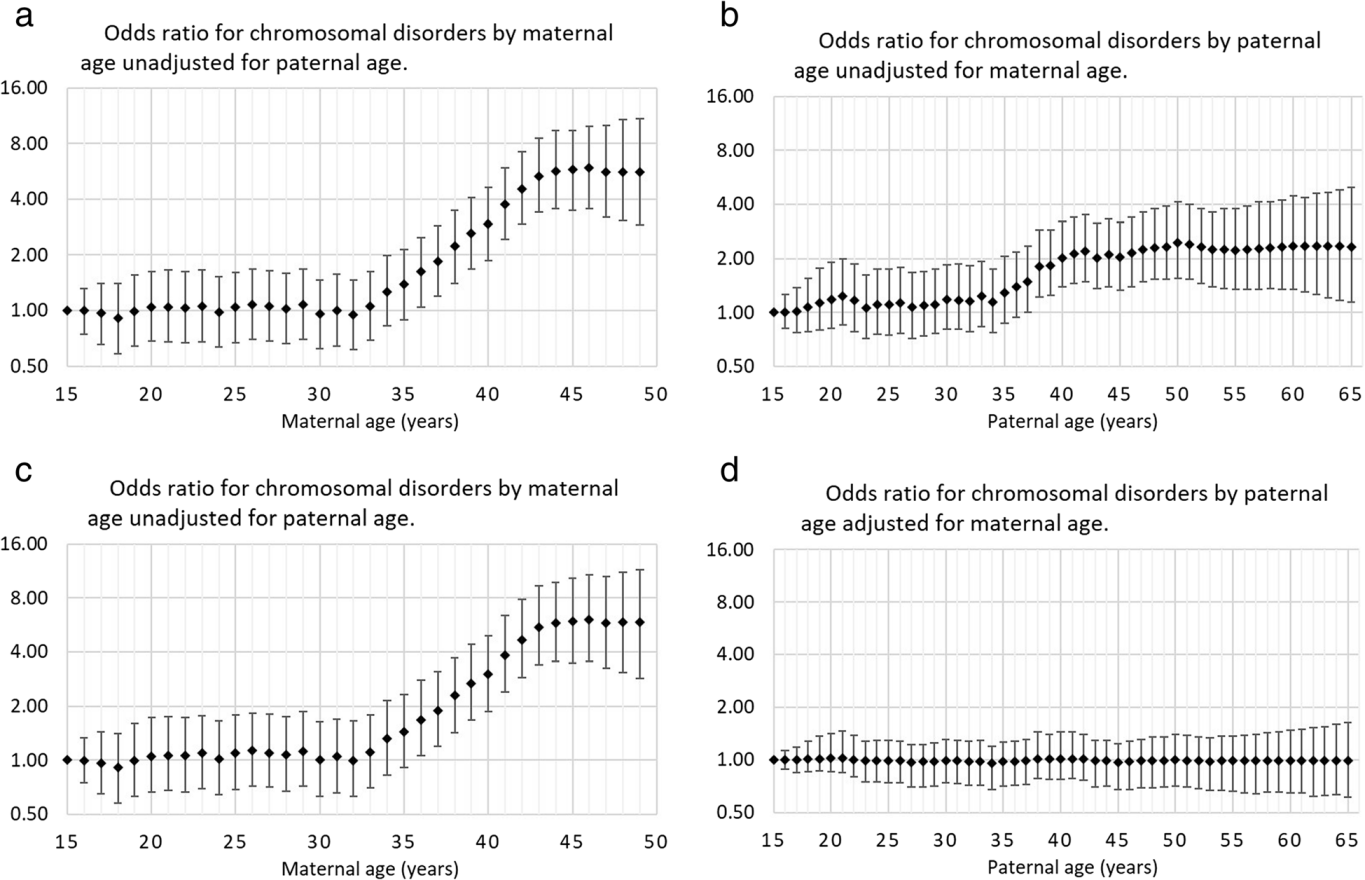
Median odds ratio and 95% Bayesian credible interval, by parental age, relative to age 15 y for chromosomal disorders (excluding Down syndrome) for (**a**) maternal age unadjusted for paternal age; (**b**) paternal age unadjusted for maternal age; (**c**) maternal age adjusted for paternal age and (**d**) paternal age adjusted for maternal age

The odds ratio for the effect of maternal age on CD, unadjusted for paternal age, started to increase at approximately age 30 and then increased relatively constantly, on the log scale, up to age 45 y, where the odds ratio appeared to stop increasing. The maximum odds ratio, relative to 15-year-old women, was approximately six-fold (Fig. 2a). The odds ratio for paternal age, unadjusted for maternal age, started to increase at approximately age 30 and then increases relatively constantly, on the log scale, up to age 45 y where the odds ratio appeared to stop increasing. The maximum odds ratio, relative to 15-year-old men, was approximately two-fold (Fig. 2b). For the effect of maternal age on CD, the plot of adjusted odds ratios was very similar to the plot of the unadjusted odds ratios. When comparing age 45 y to age 15 y, the median odds ratio and 95% credibility interval was 5.8 (3.9, 9.0) for CD (Fig. 2c). For paternal age, the plot of odds ratios showed the odds ratios for CD were very precisely near unity throughout all paternal ages. When comparing age 45 y to age 15 y, the median odds ratio and 95% credible interval was 0.98 (0.74, 1.21) for chromosomal disorders other than Down syndrome (Fig. 2d).

**Fig. 3:**
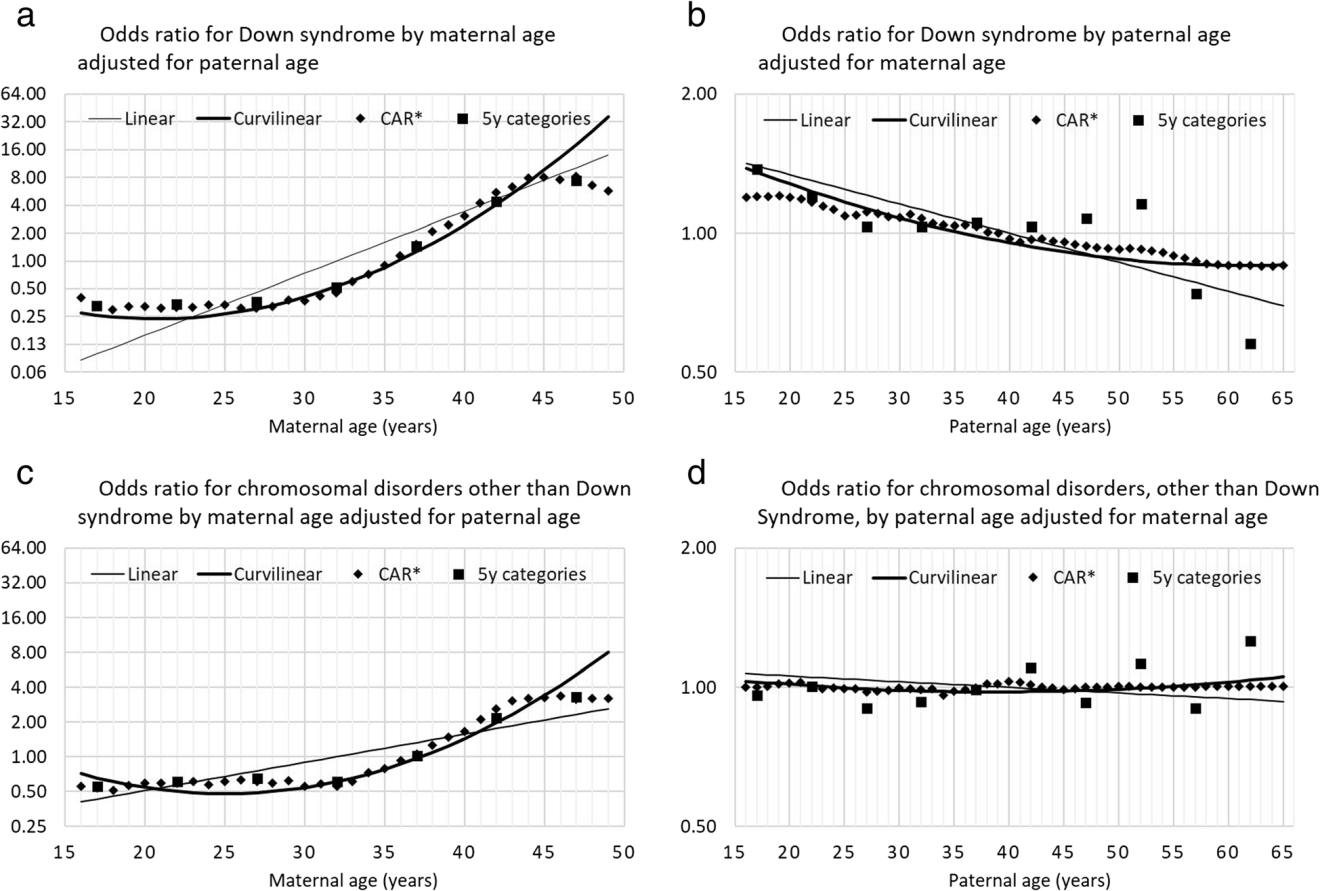
Median odds ratio, by parental age, relative to the overall mean risk for (**a**) Down syndrome by maternal age adjusted for paternal age; (**b**) Down syndrome by paternal age adjusted for maternal age and for (**c**) chromosomal disorders (excluding Down syndrome) for maternal age adjusted for paternal age and (**d**) chromosomal disorders by paternal age adjusted for maternal age. *CAR = conditional autoregressive

When comparing the final CAR models with more traditional models, all models showed strong effects of maternal age on both Down syndrome and other Chromosomal disorders (Fig. 3a and c). The linear model did not fit the other 3 models well. The curvilinear model was very similar to the CAR model and 5-year age category model up to age 45, where the CAR model and 5-year age category model both produced an inflection point. In modeling paternal effects, all models showed agreement that the effect of increasing age is related to a decrease in the birth prevalence of Down syndrome (Fig. 3b and d). None of the four models showed an association with paternal age and birth prevalence of other chromosomal disorders. For modeling the effects of paternal age, the 5-year age category model was much less smoothed than the other models, although this is shown on a very fine scale for the odds ratios. The Deviance Information Criterion (DIC) showed that the CAR model provided a far superior fit than the alternative models for the joint maternal and paternal effects (Table 1).

**Table 1:** Comparison of model fit by Deviance Information Criterion (DIC)

	Down Syndrome			Other Chromosomal Disorders		
Model	Deviance	Complexity^a^	DIC	Deviance	Complexity^a^	DIC
5-yr age category	3978.1	16.0	3994.1	3156.2	16.0	3172.2
Linear	4399.8	3.0	4402.8	3357.9	3.0	3360.9
Curvi-linear	3805.0	4.9	3809.9	3136.5	5.0	3141.5
CAR	3604.9	33.9	3638.8	3079.3	26.5	3105.8

^a^Model complexity is also referred to as number of effective parameters

## Discussion

The name “random walk prior” is used more often in time series analysis than “conditional autoregressive” but they are the same [16]. In time-series, the model is favored when long-term trends vary from linearity. The current study provides ample evidence that the maternal age function for the logit of the birth prevalence is non-linear in that the log odds does not increase uniformly each year of age. There were at least two inflection points. In comparison of the CAR model with more traditional models, the most obvious advantage of the CAR model was its ability to model multiple inflection points and rates of change for the risk. This is a result of the CAR or random walk prior being non-parametric in that there is no assumed structure among ages other than correlation among ages one year younger and one year older [16]. Non-parametric regression has been described as a preferable approach over age-band categories, fractional polynomials and spline regression but, at the time, the availability of user-friendly software was a limitation [19]. The current study used readily available software and incorporated commonly implemented and well justified prior values [20]. The OpenBUGS code and data used in the current study are provided in the supporting information. Further applications should be able to identify or resolve paternal age effects for a wide range of disorders including both childhood cancer and birth defects using existing databases.

There exists ample prior support to model maternal and paternal age effects as independent random walks for a wide variety of conditions. In females, age effects are expected to be attributed to meiosis which begins in the fetus, goes into a long period of arrested development and then is re-initiated at ovulation. Clearly, cumulative exposure would impact the arrested cells. In males, spermatagonial cells are formed by mitosis starting at puberty and, at puberty, males start a continuous process of meiosis. The sperm cells participating in fertilization began meiosis a few months before conception. While it has been reported that age-related epigenetic changes to sperm are often caused by current age-related exposures, [21] the evidence is overwhelming that, for mutations, age is acting as a surrogate for cumulative exposures of which some might be preventable. In the male, age-related, cumulative exposures will have much more impact on mitosis than meiosis. Even though faulty mitosis is considered to be more relevant in aging males, faulty paternal meiosis has been reported to cause approximately 10% of Down syndrome cases [22]. There exist important needs to identify the exposures that cause non-disjunction in oocyte development which is certainly age-related and non-disjunction in sperm development for which the role of paternal age remains unclear. From the epidemiologic perspective, the identification of the relevant risks attributable to parental ages is imperative.

For DS, the risk of maternal age did not change when controlling for paternal age. On the other hand, paternal age effects changed from very large risk to a small sparing risk when controlling for maternal age. According to a recent systematic review, a very small but statistically significant sparing effect, for paternal aging, is a novel finding [23]. In the systematic review, it was concluded that higher paternal age is probably associated with a small increase in the incidence of trisomy 21 [23]. The current study provides relatively precise risk estimates by maternal age but the risk is for birth prevalence. For Down syndrome, in the United States, both the elective termination rate and the natural loss rate are approximately 30% following diagnosis which is possible as early as 10 weeks of pregnancy and, thus, incidence (at conception) and prevalence at birth or at a time of fetal karyotyping will be very different [24]. In addition, the loss prior to 10 weeks is largely unknown but more than half normal-appearing IVF-produced embryos are aneuploidy, including often Trisomy 21 [25]. It has been shown that older women are less likely to choose elective termination with a prenatal DS diagnosis [26] but the influence of paternal age on elective pregnancy termination appears to be unknown. The very small sparing risk of advancing paternal age on birth prevalence of DS could be explained by an influence of increasing paternal age to increase the likelihood of an elective pregnancy termination. This potential bias would not be present in conditions that are not diagnosed prenatally.

## Conclusions

Advancing paternal age was not associated with an increase in risk for either Down syndrome or chromosomal disorders other than Down syndrome. For those who are familiar with Bayesian models, the proposed approach is simple to implement and interpret. Further applications are encouraged and supported.

## Additional files

Additional file 1: Model 1. Maternal Age Random-walk (CAR). OpenBUGS code and data that can be used to repeat the analyses for Model 1. (TXT 1 kb)

Additional file 2: Model 2. Paternal Age Random-walk (CAR). OpenBUGS code and data that can be used to repeat the analyses for Model 2. (TXT 2 kb)

Additional file 3: Model 3. Fully Conditional Random-walk (CAR). OpenBUGS code and data that can be used to repeat the analyses for Model 3. (TXT 67 kb)

Additional file 4: 5 year age categories. Fully conditional modeling of maternal and paternal ages as 5 year age categories. OpenBUGS code and data that can be used to repeat the analyses for the 5 year age categories. The data are arranged so that the DIC is comparable to the other fully conditional models (TXT 37 kb)

Additional file 5: Linear. Fully conditional modeling of maternal and paternal ages as linear. OpenBUGS code and data that can be used to repeat the analyses for maternal and paternal ages as linear. (TXT 65 kb)

Additional file 6: Curvilinear. Fully conditional modeling of maternal and paternal ages as curvilinear. OpenBUGS code and data that can be used to repeat the analyses for maternal and paternal ages as curvilinear. (TXT 66 kb)

## Abbreviations

CAR: Conditional autoregressive
CD: Chromosomal disorders other than Down syndrome
DS: Down syndrome
IRB: Institutional Review Board
MCMC: Markov Chain Monte Carlo
NCHS: National Center for Health Statistics
